# Embodied cognition and circular causality: on the role of constitutive autonomy in the reciprocal coupling of perception and action

**DOI:** 10.3389/fpsyg.2015.01660

**Published:** 2015-10-30

**Authors:** David Vernon, Robert Lowe, Serge Thill, Tom Ziemke

**Affiliations:** ^1^Interaction Lab, School of Informatics, University of SkövdeSkövde, Sweden; ^2^Division of Cognition and Communication, University of GothenburgGothenburg, Sweden; ^3^Human-Centered Systems, Department of Computer and Information Science, Linköping UniversityLinköping, Sweden

**Keywords:** embodied cognition, autonomy, agency, circular causality, homeostasis, allostasis

## Abstract

The reciprocal coupling of perception and action in cognitive agents has been firmly established: perceptions guide action but so too do actions influence what is perceived. While much has been said on the implications of this for the agent's external behavior, less attention has been paid to what it means for the internal bodily mechanisms which underpin cognitive behavior. In this article, we wish to redress this by reasserting that the relationship between cognition, perception, and action involves a constitutive element as well as a behavioral element, emphasizing that the reciprocal link between perception and action in cognition merits a renewed focus on the system dynamics inherent in constitutive biological autonomy. Our argument centers on the idea that cognition, perception, and action are all dependent on processes focussed primarily on the maintenance of the agent's autonomy. These processes have an inherently circular nature—self-organizing, self-producing, and self-maintaining—and our goal is to explore these processes and suggest how they can explain the reciprocity of perception and action. Specifically, we argue that the reciprocal coupling is founded primarily on their endogenous roles in the constitutive autonomy of the agent and an associated circular causality of global and local processes of self-regulation, rather than being a mutual sensory-motor contingency that derives from exogenous behavior. Furthermore, the coupling occurs first and foremost via the internal milieu realized by the agent's organismic embodiment. Finally, we consider how homeostasis and the related concept of allostasis contribute to this circular self-regulation.

## 1. Introduction

The reciprocal coupling of perception and action in cognitive agents^1^ is now well accepted and there are many examples from neuroscience and psychology, e.g., canonical visuo-motor neurons (Rizzolatti and Fadiga, 1998), mirror neurons (Rizzolatti et al., 1996; Rizzolatti and Craighero, 2004; Thill et al., 2013), and a variety of ways in which embodiment influences perceptual, motor, and cognitive performance (Varela et al., 1991; Barsalou et al., 2003). However, cognition is more than a collection of perceptuo-motor contingencies. In Varela's words, cognition is *effective action* (Maturana and Varela, 1987; Varela et al., 1991): action that preserves the agent's autonomy, maintaining the agent and its ontogeny, i.e., its continued development. Prospection, i.e., prediction or anticipation, is one of the two hallmarks of a cognitive agent, the second being the ability to learn new knowledge by making sense of its interactions with the world around it and, in the process, enlarging its repertoire of effective actions (Vernon, 2010; Vernon et al., 2010). Cognition entails being able to anticipate the need for action and being able to anticipate the outcome of that action. According to some theories, e.g., Hesslow's simulation hypothesis (Hesslow, 2002, 2012), this can be achieved with a form of internal simulation focusing on goal-directed prospective action selection and adaptation. Perhaps the best encapsulation of this prospective goal-directed perceptuo-motor approach is ideomotor theory (Stock and Stock, 2004).

**Figure 1:**
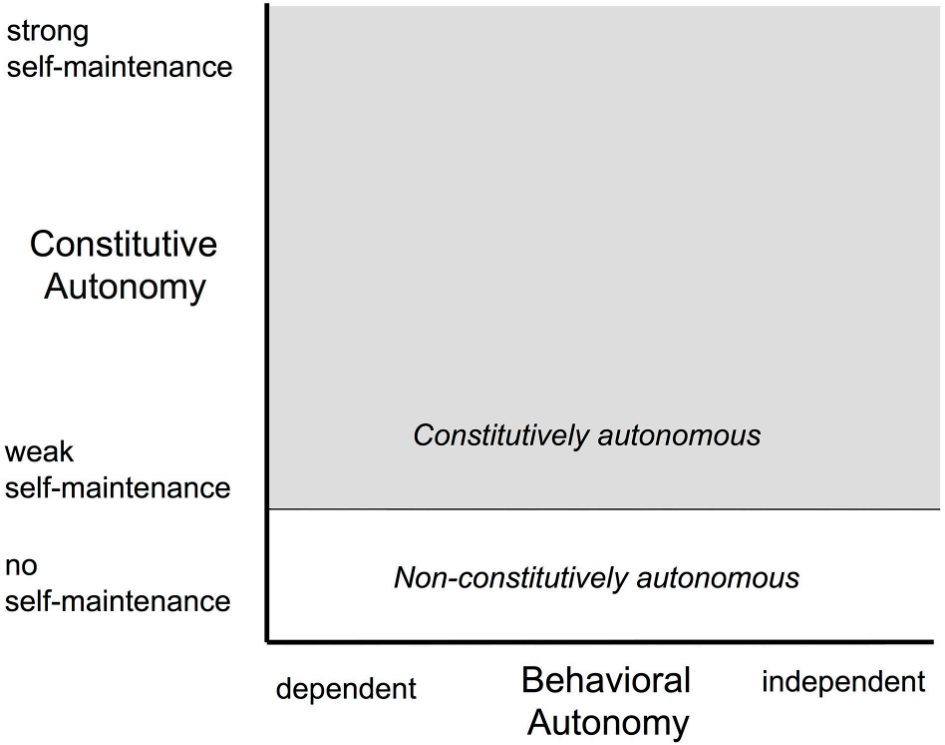
**A characterization of autonomous agents situated in a two-dimensional space spanned in one dimension by behavioral autonomy and in the other by constitutive autonomy (based on Froese et al., 2007)**. The behavioral dimension focusses on the degree of independence of human assistance and the extent to which the system sets its own goals, and therefore corresponds loosely to what is referred to as the *degree of autonomy* in robotics. The constitutive autonomy dimension focusses on the organizational characteristics that allow the system to maintain itself as an identifiable autonomous entity. Since some systems don't exhibit the requisite organizational characteristics (e.g., organizational closure; see main text), they aren't constitutively autonomous. These occupy the white region at the bottom of the space. Those systems that *are* constitutively autonomous can make different levels of contribution to the maintenance of their autonomy and, thus, this dimension corresponds loosely to *strength of autonomy* and the *task entropy* in robotics (Sheridan and Verplank, 1978).

Such a characterization, however, while necessary and useful, ascribes only behavioral attributes to cognition. We wish to reassert here that cognition also has a constitutive aspect, one that complements the behavioral aspect. The constitutive/behavioral distinction derives from the constitutive autonomy and behavioral autonomy of biological agents (Froese et al., 2007; Froese and Ziemke, 2009), especially those systems that exhibit the characteristic of recursive self-maintenance (Bickhard, 2000), capacities that reflect Varela's and Maturana's concepts of autopoiesis (Maturana, 1970, 1975; Maturana and Varela, 1980), organizational closure (Varela, 1979; Maturana and Varela, 1987), and operational closure (Froese and Ziemke, 2009; Stewart et al., 2010); see Figure 1. In this view, cognition, perception, and action serve to support the autonomy of the agent, both in a constitutive sense and in a behavioral sense. The constitutive aspect of autonomy focusses on the dynamic self-organization of the agent as an embodied system, maintaining itself as a viable structure that can support the organizational processes in the first place. The behavioral aspect of autonomy, on the other hand, targets the interaction of that embodied system with the environment in which it is embedded, maintaining the external conditions which are necessary for constitutive autonomy. We develop further the issue of constitutive autonomy in Section 2 on autonomy and deal with it more definitively in Section 3 on constitutive processes.

The goal of this article is to argue the case that the reciprocal coupling of action and perception is founded primarily on their roles in the constitutive autonomy of the agent and an associated circular causality of global and local processes of self-regulation, rather than being a mutual sensory-motor contingency that derives from exogenous behavior. Our objective is the synthesis of three strands of thinking into a cohesive picture: (a) the distinction between constitutive and behavioral autonomy and related processes, (b) the dynamics of circular causality, and (c) allostatic self-regulation (in contradistinction to homeostatic self-regulation). While all of these strands can indeed be traced to previous work, this paper is, to our knowledge, the first such synthesis.

The article begins with a discussion of biological autonomy, clarifying the distinction between constitutive and behavioral autonomy. This sets the scene for the introduction of constitutive processes. We begin with an explanation of the difference between self-organization and emergence and then summarize the key processes of autopoiesis, organizational closure, and structural coupling. These processes exhibit the pivotal attributes of continuous reciprocal causation and circular causality. We consider how homeostasis and the related concept of allostasis contribute to this circular self-regulation. Finally, we explain how the reciprocal coupling of perception and action can be understood in this framework, arguing that the coupling happens first and foremost via the internal milieu realized by the agent's embodiment. In presenting this synthesis, the article integrates and builds on many quite disparate concepts, not all of which will be familiar to every reader; an introduction to many of these ideas can be found in tutorial texts (e.g., Vernon, 2014).

## 2. Autonomy

Autonomy is a difficult concept to tie down (Boden, 2008) and there are several perspectives on what it means (Froese et al., 2007). For convenience, we will adopt a definition of autonomy as the degree of self-determination of a system, i.e., the degree to which a system's behavior is not determined by the environment and, thus, the degree to which a system determines its own goals (Ziemke, 1997, 1998; Bertschinger et al., 2008; Seth, 2010; Vernon, 2014). Implicit in this definition is the idea that, in addition to selecting its goals, the agent can then choose how best to achieve them and that it can then act to do so. For biological autonomous entities, the issue of autonomy becomes one of survival, typically in the face of precarious conditions, i.e., environmental conditions in which the entity has to work to keep itself alive as an autonomous system, both physically and organizationally as a dynamic self-sustaining entity.

Living systems face two problems: they are delicate and they are dissipative. Being delicate means that they are easily disrupted and possibly destroyed by the stronger physical forces present in their environment (including other biological agents). Consequently, living systems have to avoid these disruptions and repair or heal them when they do occur. Dissipation arises from the fact that living systems are comprised of far-from-equilibrium processes (Bickhard, 2000). This means that the system must have some external source of energy or matter if they are to avoid lapsing into a state of thermodynamic equilibrium. If they do succumb to this, they come to rest and cease to be able to change in response to or in anticipation of any external factors that would threaten their autonomy or their existence^2^. Again, as with the delicacy of living systems, the dissipation inherent in far-from-equilibrium stability means that the system has to continually acquire resources and repair damage to itself. All of this has to be done by the agent itself. Of course, it is better if the agent can avoid damage in the first place and cognition as a prospective modulator of perception and action is one of the primary mechanisms at the agent's disposal (Barandiaran and Moreno, 2008). By bringing to bear a capacity for prospection, cognition compensates for the fact that perception is bound to the here-and-now and allows the agent to anticipate the need for action and the outcome of that action.

From this perspective, autonomy, aided by cognition, *is* the self-maintaining organizational characteristic of living creatures that enables them to use their own capacities to manage their interactions with the world in order to remain viable: i.e., compensate for dissipation, avoid disruption, and self-repair when necessary (Christensen and Hooker, 2000). In other words, autonomy is the process by which a system manages—self-regulates—to maintain itself as a viable entity in the face of the precarious circumstances with which the environment continually confronts it. In Bickhard's words “the grounds of cognition are adaptive far-from-equilibrium autonomy—recursively self-maintenant autonomy” (Bickhard, 2000).

One can distinguish two types of autonomy: *behavioral autonomy* and *constitutive autonomy* (Froese et al., 2007; Barandiaran and Moreno, 2008; again, refer to Figure 1). Behavioral autonomy focusses on the external characteristics of the system: the extent to which the agent sets its own goals and its robustness and flexibility in dealing with an uncertain and possibly precarious environment. On the other hand, constitutive autonomy focusses on the internal organization and the organizational processes that keep the system viable and maintain itself as an identifiable autonomous entity. Agents that are constitutively autonomous can make different levels of contribution to the maintenance of their autonomy, making them less or more effective in dealing with the uncertainty and precariousness of the environment in which the system is embedded and in which it has to survive. Behavioral and constitutive autonomy are linked: an agent can't deal with uncertainty and danger if it is not organizationally—constitutively—equipped to do so. Its behavior depends on internal preparedness, achieved both through the neural mechanisms of the central and peripheral nervous system and the hormonal mechanisms of the endocrine system. On the other hand, in precarious circumstances, the agent needs behavioral autonomy to allow it to achieve the requisite environmental conditions—through interaction—for constitutive autonomy to be able to operate at all. This complementarity of the constitutive and the behavioral reflects two different sides of the characteristic of recursive self-maintenant systems to deploy different processes of self-maintenance depending on environmental conditions: one—constitutive autonomy—is the internal endogenous aspect of that adaptive capacity and the other—behavioral autonomy—is the external exogenous aspect of that adaptive capacity.

The constitutive-behavioral distinction is sometimes cast as a difference between *constitutive* processes and *interactive* processes (Froese and Ziemke, 2009). As we have said, constitutive processes deal with the agent itself, its organization, and its maintenance as an agent through on-going processes of self-construction and self-repair. On the other hand, interactive processes deal with the interaction of the agent with its environment. Both processes play complementary roles in autonomous operation of the agents. Constitutive processes are more fundamental to the autonomy of the agent but both are required.

## 3. Constitutive processes

### 3.1. Self-organization vs. emergence

Autonomy is closely linked to self-organization. Boden notes that autonomy, self-organization, and freedom are three notoriously slippery notions and none of them can be properly understood without considering the others (Boden, 2008). One definition of self-organization goes as follows.

“A process in which pattern at the global level of a system emerges solely from numerous interactions among the lower-level components of the system. The rules specifying interactions among the system's components are executed using only local information, without reference to the global pattern”

(Camazine, 2006).

Emergence also refers to a process involving interacting components in a system and the consequent generation of a global pattern. However, in this case, the global pattern emerges as something qualitatively different from the underlying assembly of components and, most significantly, is not simply a consequence of the superposition of the contributions of the individual components^3^.

The form of self-organization in emergence gives rises to systems that have a clear identity or behavior that results from two factors: (a) local-to-global determination and (b) global-to-local determination. In local-to-global determination, the emergent process has its global identity constituted and constrained by local interactions. In global-to-local determination, the global identity and its interaction with the system environment constrain the local interactions (Thompson and Varela, 2001; Froese and Ziemke, 2009; Di Paolo et al., 2010). This is sometimes referred to as *emergent self-organization*. Such self-organization has also been defined as “the spontaneous emergence (and maintenance) of order, out of an origin that is ordered to a lesser degree” (Boden, 2008). This definition provides the key link between self-organization, emergence, and autonomy: that self-organization results from the intrinsic spontaneous character of the system (possibly involving interaction with the environment) rather than being imposed by some external force or agent. In other words, emergent self-organization is autonomous and, *vice versa*, autonomous systems typically involve some form of emergent self-organization.

Emergent self-organization gives rise to a special view of autonomy, a view that is also characterized by *self-production*. Not only is there a reciprocal local-global and global-local determination but the nature of the determination is to re-create the local components from which the global system arises. This is constitutive autonomy (Froese and Ziemke, 2009). A system which exhibits constitutive autonomy actively generates and sustains its existence and systemic identity under precarious conditions, i.e., conditions that are antagonistic to the delicate and dissipative nature of the cognitive agent and which, in the absence of some appropriate form of emergent self-organization and associated behavior, would cause the system to cease to exist and cause its identity to be destroyed.

### 3.2. Self-production and self-construction: autopoiesis and organizational closure

Constitutive autonomy is related to the concept of *organizational closure*. Varela famously equates autonomy with organizational closure:

“Autonomous systems are mechanistic (dynamic) systems defined as a unity by their organization. *We shall say that autonomous systems are organizationally closed. That is, their organization is characterized by processes such that (1) the processes are related as a network, so that they recursively depend on each other in the generation and realization of the processes themselves, and (2) they constitute the system as a unity recognizable in the space (domain) in which the processes exist*”

(Varela, 1979, p. 55; emphasis in the original).

Maturana and Varela subsequently define autonomy as “the condition of subordinating all changes to the maintenance of the organization” (Maturana and Varela, 1980).

Organizational closure is a necessary characteristic of a particular form of self-producing self-organization called *autopoiesis* (Maturana, 1970, 1975) that operates at the bio-chemical level, e.g., in cellular systems. Autopoietic systems are quite literally self-organizing systems that self-produce. Maturana and Varela later expanded the concept to deal with autonomous systems in general and refer to it in this context as *operational closure*, rather than autopoiesis which is specific to the bio-chemical domain. The operational closure vs. organizational closure terminology can be confusing because in some earlier publications (e.g., Varela, 1979), Varela refers to *organizational closure* but in later works (by Maturana and Varela themselves, e.g., Maturana and Varela, 1987, and by others, e.g., Stewart et al., 2010) this term was subsequently replaced in favor of *operational closure*. However, the term operational closure is appropriate when one wants to identify any system that is identified by an observer to be self-contained and parametrically coupled with its environment but not controlled by the environment. On the other hand, organizational closure characterizes an operationally-closed system that exhibits some form of self-production or self-construction (Froese and Ziemke, 2009).

These organizational principles are also reflected in the concepts of Bickhard's *self-maintenance* and *recursive self-maintenance* in far-from-equilibrium systems (Bickhard, 2000). Arguably, these two concepts represent a generalization of the ideas of self-construction and self-production introduced by Maturana and Varela in their processes of autopoiesis, organizational closure, and operational closure. Self-maintenant systems contribute to the conditions which are necessary to maintain it, i.e., to keep it going. In contrast, recursive self-maintenant systems exhibit a stronger form of autonomy in that they can deploy different processes of self-maintenance depending on environmental conditions, recruiting different self-maintenant processes as conditions in the environment require. Self-maintenance and recursive self-maintenance align well with the concepts of self-organization and emergent self-organization (both constitutive and behavioral autonomy), respectively.

### 3.3. Continuous reciprocal causation and circular causality

In the foregoing, there has been a recurring theme: a circular relationship between part and whole: between local factors and global factors. It appears that the characteristics of emergence and emergent self-organization are dependent on dynamic re-entrant structures. This is related to the concept of *continuous reciprocal causation* (CRC; Clark, 1997) which occurs when some system is both continuously affecting and simultaneously being affected by activity in some other system (Clark, 1998)^4^. In effect, one system causes an effect in a second system which then causes an effect in the first, reinforcing the dynamic and causing the process to continue. CRC can also occur in a single system. In this case, the causal contribution of each systemic component partially determines, and is partially determined by, the causal contributions of large numbers of other systemic components. Wheeler puts it like this: “CRC is causation that involves multiple simultaneous interactions and complex dynamic feedback loops, such that (a) the causal contribution of each systemic component partially determines, and is partially determined by, the causal contributions of large numbers of other systemic components, and, moreover, (b) those contributions may change radically over time” (Wheeler, 2008).

This single-system CRC is often referred to as *circular causality* or *circular causation* (Varela, 1979). While circular causality can occur between distinct sub-systems in this overall system, it more usually reflects the interaction between global system dynamics (the whole) and local system dynamics (the parts). For example, Kelso uses the term circular causality to describe the situation in dynamical systems where the cooperation of the individual parts of the system determines the global system behavior which, in turn, governs the behavior of these individual parts (Kelso, 1995). Thus, circular causality exists *between* levels of a hierarchy of system and sub-system. This influence of macroscopic levels on microscopic levels in a system is captured in the term *downward causation* i.e., that global-to-local or macroscopic-to-microscopic aspect of circular causality whereby the global system behavior causally influences the individual system components (Thompson and Varela, 2001; Seth, 2010). In circularly causal systems, global system behavior influences the local behavior of the system components and yet it is the local interaction between the components that determines the global behavior. Thus, in biological autonomy, the degree of participation of the components of a system is determined by the global behavior which, in turn, is determined by the interactions among the components through causal reciprocal feedback loops. Again, these ideas are also echoed in Bickhard's concept of recursive self-maintenance (Bickhard, 2000).

The idea of circular causality is also related to the notion of entrainment where the global macro state entrains the micro-constituent processes of which it comprises in order to maintain that macro state. This has been applied to higher level forms of constitutive processing, e.g., in human emotions, where the macro states provide a substrate for learning (Lewis, 2005). Similarly, circular causality is a central feature of an information-theoretic model of self-sustainability, i.e., autonomy, in ecosystem networks (Ulanowicz, 1998, 2000, 2011). The same model has been used to characterize the emergence and development of beliefs in human cognition (Castillo et al., in press). This network-centric perspective aligns with the interaction-dominant view of cognitive dynamics which highlights that cognition is characterized by interactions among multiple spatial and temporal scales of organization and among nested structures, rather than by relationships between simpler components at a single scale (i.e., the component-dominant view; Ihlen and Vereijken, 2010; Dixon et al., 2012).

## 4. Realizing circular causality

How might circular causality be manifest in a cognitive agent and, more generally, in a system that exhibits constitutive autonomy? In this section, we consider how homeostasis and the related concept of allostasis contribute to circular global-to-local and local-to-global self-regulation.

### 4.1. Homeostasis

The process of self-regulation is central to constitutive autonomy. In biological systems, the automatic regulation of physiological functions is referred to as *homeostasis*, a term coined by Cannon (1929) formalizing the idea advanced in the nineteenth century by Claude Bernard that “all the vital mechanisms, however varied they may be, have only one object, that of preserving constant the conditions of life in the internal environment" (Bernard, 1878). Put simply, homeostatic processes regulate the operation of a system in order to keep the value of some system variables constant or within acceptable bounds, e.g., body temperature and blood glucose level. It does this by sensing any deviation from the desired value and feeding this error back to the control mechanism to correct the error. The desired value is called the *setpoint* in control theory and the use of the deviation from the desired value is called feedback.

We have in previous work (Morse et al., 2008; Ziemke and Lowe, 2009) suggested that the autonomy of an agent is effected through a hierarchy of homeostatic self-regulatory processes, exploiting a spectrum of associated affective (i.e., emotional or feeling) states, ranging from basic reflexes linked to metabolic regulation, through drives and motives, and on to the emotions and feelings often linked to higher cognitive functions. The progression of processes of homeostasis from basic reflexes and metabolic regulation, through drives and motives, to emotions and feelings is described in a schema for a cognitive architecture that places affect on an equal footing with more conventional cognitive processes. This progression follows closely Damasio's hierarchy of levels of homeostatic regulation (Damasio, 2003) and is based on a relatively broad notion of homeostasis as “the process of maintaining the internal milieu physiological parameters (such as temperature, pH and nutrient levels) of a biological system within the range that facilitates survival and optimal function” (Damasio, 2003; Damasio and Carvalho, 2013). Different homeostatic processes regulate different system properties.

Typically, the autonomous agent is perturbed during interactions with the world with the result that the organizational dynamics have to be adjusted. This process of adjustment is exactly what is meant by homeostasis— self-regulation—and the motives at every level of this hierarchy of homeostatic processes are effectively the drives that are required to return the agent to a state where its autonomy is no longer threatened. In the interaction with the world around it, the perturbations of the agent by the environment have no intrinsic value in their own right—they are just the stuff that happens to the agent as it goes about its business of survival—but for the agent this stuff, these interactions and perturbations, have a perceived value in that they act to endanger or support its autonomy. This value is conveyed through the affective aspect of these homeostatic processes and consequently the agent then attaches some value to what is an otherwise neutral world (even if it is a precarious one; Di Paolo, 2005). The implications for perception and action are significant and this brings us to the crucial issue regarding the reciprocal coupling of action and perception in cognition.

First, perceptions and actions form a complementary set of environment-agent/agent-environment perturbations that are related not as extrinsic stimulus-response perceptuo-motor contingencies but as intrinsic processes that lead to the regulation of the system and autonomy preservation through emergent self-organization. The processes of perception and action are mutually dependent because they are both modulated by the system—globally-determined—through downward causation and, together with other homeostatic processes, they give rise to the global constitutive autonomy-preserving system behavior.

Second, perception and action are reciprocally coupled and mutually dependent because, from the perspective of enactive cognitive science, perception and action form a joint process of making sense of the world in which the agent is embedded (Maturana and Varela, 1987; Varela et al., 1991; Vernon, 2010). This “sense” captures the lawfulness of the agent's environment as it relates to the agent's constitutive and behavioral autonomy. Since the agent is an organizationally closed system, perception and action are perturbing forces rather than system inputs and outputs. This process of mutual perturbation of the agent and environment in which it is embedded, facilitating the on-going operational identity of the agent and its autonomous self-maintenance, is known as *structural coupling*. The process of structural coupling produces an embodiment-specific congruence between the system and its environment. For this reason, we say that the system and the environment are *co-determined*. In enactive cognitive science, this is also referred to as *structural determination* to emphasize the dependence of an agent's space of viable environmentally-triggered changes on the agent's structure, i.e., its particular embodiment, and its internal dynamics (Maturana and Varela, 1987; Varela et al., 1991).

### 4.2. Allostasis

While many autonomous agents are self-governing in the sense that they adjust automatically to events in the environment and self-correct when necessary (e.g., by way of homeostasis), other autonomous agents begin to adjust *before* the event actually occurs. This form of autonomy requires a continual preparation for what might be coming next. It means that an autonomous system anticipates what events might occur in its environment and actively prepares for them so that it is capable of dealing with them if they do occur. From this perspective, autonomy requires pre-emptive action, not just reactive action, and predictive self-regulation, not just reactive self-regulation. These autonomous systems ready themselves for multiple contingencies, i.e., possible events, and have several strategies for dealing with them. They deploy them while pursuing some goal or other that the system has defined for itself. To an extent, this characteristic can be viewed as predictive self-regulation and is known as *allostasis* (Sterling, 2004; Schulkin, 2011; Sterling, 2012).

Allostasis encourages a rethink of the classical control theoretic perspective on homeostasis revolving around feedback loops respecting set points that demarcate ideal states. According to Sterling (Sterling, 2004), allostasis can be conceived in terms of prediction where brain areas implicated in planning and decision making are viewed as supplying inputs that may override other inputs that signal errors from ideal homeostatic balance. Such global overriding of “basal” homeostasis operates in the service of supplying the organism with the resources previously learned to be necessary to meet predicted environmental pressures. Sterling considers allostasis as a means of permitting adaptive bodily regulation according to “stability through change” which accounts for both internal needs and external pressures (or opportunities) in contrast to the Bernard notion of “stability through constancy.” Thus, allostasis is concerned with adapting to change in order to achieve the goal of stability in the face of uncertain circumstances. Efficient regulation requires the anticipation of needs and preparation to satisfy them before they arise: “The brain monitors a very large number of external and internal parameters to anticipate changing needs, evaluate priorities, and prepare the organism to satisfy them *before* they lead to errors. The brain even anticipates its own local needs, increasing flow to certain regions—before there is an error signal” (Sterling, 2012). For example, human behavior in adapting to pain involves such predictive regulation, rather than mere reaction to tissue damage; that means “the nervous system is organized to anticipate potential pain and to adjust behavior before the risk of tissue damage becomes critical” (Morrison et al., 2013).

Allostasis, rather than being based on a reciprocal sharing of resources among systems (classic homeostasis), entails a degree of centralized control over sub-systems (Sterling, 2012). This can also be viewed in terms of downward causation: while Lewis (2005) references the macroscopic state that entrains its “emotion”-based microscopic constituents in the service of learning, Sterling (2012), p. 14, refers to “[memory] retrieval involv[ing] elaborate connections within “limbic” structures …that …project in cascades to prefrontal cortex.” The limbic (emotion) system's constituents subserve constitutive organization (they relay signals related to sustaining the viability of the organism) and may be entrained by prefrontal cortex (which also reciprocally connects to neocortex) to facilitate adaptive behavior over a goal-directed sequence.

The focus on predictive regulation in allostasis mirrors strongly the anticipatory nature of cognition. Seth (2013) emphasizes this by developing the role that interoceptive predictive coding (as distinct from the more usual view of prospection in exteroceptive predictive coding) plays in the experience of “body ownership and conscious selfhood,” viewing emotions—subjective feeling states—as emerging from cognitive evaluations of physiological changes. As such, he targets a larger quarry in cognitive science: consciousness and neuropsychiatric illness. He hypothesizes that predictive coding arises through “an extended autonomic neural substrate” (Seth, 2013), p. 565, taking the principle on which allostasis is based—the causal role played by prediction in biological regulation—to the next level. Specifically, he highlights the role of active inference, an extension of predictive coding, whereby the interoceptive prediction errors can be suppressed not by updating the generative model that gave rise to the predictions but by internal action, translating the predictions into reference points for autonomic regulatory processes, e.g., physiological homeostasis. He notes that attention can then be viewed as a way of balancing active inference and model update, (referred to as precision weighting). Seth reinforces the idea that “an organism should maintain well-adapted predictive models of its own physical body …and of its internal physiological condition” (Seth, 2013), p. 567, and in Seth (2015) he develops this further, grounding predictive coding, active infererence, and the principle of free energy in cybernetics and allostatic mechanisms for the maintenance of internal organization. Barrett and Simmons (2015) emphasize the same point with their *Embodied Predictive Interoception Coding* (EPIC) model, pointing out the direct link between active inference in the cortex and interceptive predictions of the internal milieu of the body, i.e., its physiological state relating to, e.g., heart rate, glucose levels, carbon dioxide in the bloodstream, and temperature.

In summary, allostasis differs from homeostasis in its predictive character and in its ability to anticipate and adapt to change rather than resist it. Significantly, allostasis is effected at a higher level of organization, involving greater number of sub-systems acting together in a coordinated manner with global processes modulating local ones, reflecting the character of circular causality. In contrast, mechanisms for homeostasis operate at a simpler level of negative feedback control (Sterling, 2004; Muntean and Wright, 2007; Sterling, 2012). Although you can view allostasis as a complementary mechanism to homeostasis, Sterling notes that it was introduced as a potential replacement for homeostasis as the core model of physiological regulation (Sterling, 2004, 2012).

In the next section, we look briefly at one example of how the principles of homeostasis and allostasis can be used to describe how cognition arises through constitutive autonomy.

### 4.3. Toward a constitutive autonomy cognitive architecture

Based on Damasio's view of the architecture and physiology of the mammalian brain, we have in previous work proposed two schemas for an enactive cognitive architecture that explicitly embraces the constitutive-behavioral distinction (Morse et al., 2008; Ziemke and Lowe, 2009). They are schemas in the sense that they identify the principal characteristics of the architecture without providing a detailed design of the component parts of the architecture and the dynamics of their interaction. A design approach called *holistic-reductionism* complements the schemas, focussing on the interdependencies of the components rather than on the identification of independent functional modules, as is normally the case with computational cognitive architecture design. Any modularity in the system emerges from the interdependence of the embodied cognitive processes rather than by phylogenetic pre-specificiation.

The first version of the architecture schema traverses two dimensions: (i) Constitutive Organization and (ii) Behavioral Organization (Morse et al., 2008). The former refers to the system's internal dynamics as it maintains its integrity—its autonomy—in the face of perturbation by various stimuli. At the core of this space there is metabolic homeostatic self-regulation. This extends to stimulus valence evaluation, somatic state response, and content evaluation, each level offering increasing organizational complexity, an increasing degree of decoupling between stimulus and response, and an increasing degree of appraisal and associated adaptivity. Each level in the constitutive organization dimension is matched by an associated level in the behavioral organization dimension: approach-avoidance, sequenced behavior, and multi-sequenced behavior, respectively. Thus, the behavioral organization dimension is coupled by sensorimotor perception to the constitutive organizational dimension.

**Figure 2:**
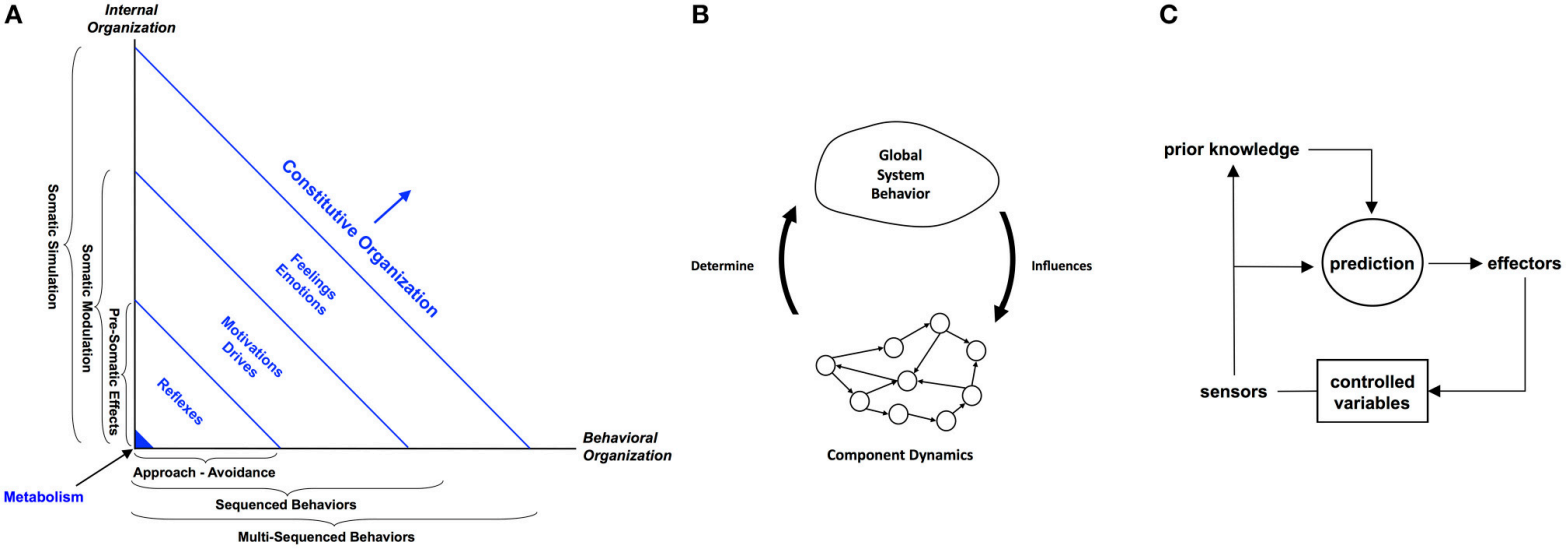
**The three strands of thought being advanced in this paper are (A) the distinction between constitutive and behavioral autonomy and related processes, (B) the dynamics of circular causality, and (C) predictive allostatic self-regulation**. The Cognitive-Affective Architecture Schematic in **(A)** is an example of the first aspect. It exhibits a spectrum of constitutive organization brought about by the recruitment of a progression of emotions, from reflexes, through drives and motivations, to emotions-proper and feelings. Each level in the constitutive organization is associated on the Internal Organization axis with an increasing level of homeostatic autonomy-preserving self-maintenance, ranging from basic metabolic processes through reactive sensorimotor activity (pre-somatic effects), associative learning and prediction (somatic modulation), to interoception and internal simulation of behavior prior to action. Equally, each level in the constitutive organization is associated on the Behavioral Organization axis with an increasing level of complexity in behavior, ranging from approach-avoidance, sequenced behaviors, and multi-sequenced behaviors. A more complete cognitive architecture that fully embraces constitutive autonomy would also incorporate processes for circular causality and allostasis.

A later version of the architecture (see Figure 2) reflects this coupling by referring to a single space of constitutive organization which is viewed from two perspectives: internal organization and behavioral organization (Ziemke and Lowe, 2009). The spectrum of constitutive organization is realized by the recruitment of a progression of emotions, from reflexes, through drives and motivations, to emotions-proper and feelings. Each level in constitutive organization is associated on the internal organization axis with an increasing level of homeostatic autonomy-preserving self-maintenance, ranging from basic metabolic processes through reactive sensorimotor activity (pre-somatic effects), associative learning and prediction (somatic modulation), to interoception and internal simulation of behavior prior to action. Equally, each level in constitutive organization is associated on the behavioral organization axis with an increasing level of complexity in behavior, ranging from approach-avoidance, sequenced behaviors, and multi-sequenced behaviors.

The key idea is that different levels of cognitive function and behavioral complexity are associated with, and are brought about by, different levels of emotion, each linked to affective homeostatic processes ranging from reflexes right through to internal simulation. An extension of this schematic that augmented the homeostatic processes with allostatic ones would embrace more fully the concepts of constitutive autonomy advanced in this paper, including, as we have mentioned above, circular causality.

## 5. Conclusion

Cognition is commonly cast as a prospective process of adaptation, growth, and development (Vernon, 2010; Vernon et al., 2010), often focussing on behavioral autonomy. Here, however, we have recast cognition in a different light that emphasizes the importance of constitutive autonomy. Specifically, we have argued for a form of predictive regulation—allostasis—that is intrinsic to adaptive agents and exhibits a type of circular causality that naturally gives rise to reciprocal coupling of perception and action in embodied agents. The resulting synthesis of these different lines of thinking lead us to argue that perceptions and actions form a complementary set of environment-agent/agent-environment perturbations that are related not only as extrinsic stimulus-response perceptuo-motor contingencies but also as intrinsic processes that lead to the regulation of the system and autonomy preservation through emergent self-organization. The perceptions and actions are mutually dependent because they are both modulated by the system—globally-determined—through downward causation. Together they form a process of mutual perturbation of the agent and environment in which it is embedded, i.e., structural coupling, that facilitates both constitutive and behavioral autonomy. It remains as a significant research challenge to uncover the specific mechanisms by which circular causality and allostasis arise in natural agents and how—and to what degree—they might be replicated in artificial systems.

### Conflict of interest statement

The authors declare that the research was conducted in the absence of any commercial or financial relationships that could be construed as a potential conflict of interest.

## Footnotes

^1^We here use the term *agent* to refer to any system that displays a cognitive capacity, whether it is a human or and artificial cognitive system, such as a cognitive robot.

^2^Self-healing structure-maintaining properties are also exhibited by some non-living non-equilibrium dissipative entropy-producing complex systems (Kondepudi et al., 2015).

^3^Seth distinguishes between nominal, weak, and strong emergence (Seth, 2010). Nominal emergence is simply the idea that emergence is some property that can be exhibited by a complete macro-level object or system but not by its constitutent parts. Strong emergence claims that macro-level properties are in principle not deducible from observation of the micro-level components and they have causal powers that are irreducible, i.e., they arise only because of the existence of the emergent behaviors. These causal powers are directed at the behavior of the components from which the emergent pattern emerges. Weak emergence sits somewhere in between nominal and strong emergence. It doesn't commit to the principled irreducibility of macroscopic patterns or behavior to microscopic activity but asserts that the relationship between the two levels is complex.

^4^Reciprocal causation (Laland et al., 2012) also exists between proximate and ultimate mechanisms of evolutionary biology (Mayr, 1961; Tinbergen, 1963; Scott-Phillips et al., 2011).
